# 干细胞转录因子Sox2在肺癌中的表达和意义

**DOI:** 10.3779/j.issn.1009-3419.2013.11.05

**Published:** 2013-11-20

**Authors:** 伟 许, 云艳 位, 瑶曦 谭, 玮 徐, 雁 程, 剑卿 吴

**Affiliations:** 1 210029 南京，南京医科大学第一附属医院老年呼吸科 Department of Geriatrics, the First Affilicated Hospital of Nanjing Medical University, Nanjing 210029, China; 2 210009 南京，江苏省肿瘤医院病理科 Department of Pathology, Jiangsu Cancer Hospital, Nanjing 210029, China; 3 210029 南京，南京医科大学发育遗传学系 Department of Developmental Genetics, Nanjing Medical University, Nanjing 210029, China

**Keywords:** 肺肿瘤, Sox2, Sox2自身抗体, 肿瘤干细胞, Lung neoplasms, Sox2, Sox2 self-antibody, Cancer stem cells

## Abstract

**背景与目的:**

转录因子Sox2维持干细胞的全能性，参与肿瘤干细胞的自我更新，在多种肿瘤的发生发展中发挥重要作用。本研究旨在探讨Sox2及Sox2自身抗体（Sox2-Ab）在非小细胞肺癌（non-small cell lung cancer, NSCLC）患者组织及血清中的表达和意义。

**方法:**

荧光定量PCR及免疫组化法检测58例NSCLC、16例其他肿瘤和20例正常肺组织标本中*Sox2*基因及蛋白表达，ELISA法检测30例NSCLC患者和30例健康体检者血清Sox2-Ab水平，结合NSCLC临床病理特点进行数据分析。

**结果:**

肺癌组织中Sox2 mRNA水平及蛋白阳性表达率均高于其他肿瘤及正常肺组织，差异均有统计学意义（*P* < 0.01），且Sox2 mRNA表达增高同肺癌患者病理类型及肿瘤大小有关，与性别、年龄、肿瘤分化程度和淋巴结转移等无关。血清Sox2-Ab水平在NSCLC患者和正常体检者差异无统计学意义。

**结论:**

Sox2在NSCLC中有较高的表达，与病理类型、肿瘤大小密切相关，Sox2可能成为肺癌新的标志物及治疗靶点。

## Abstract (en)

**Background and objective:**

Transcription factor Sox2 remains the pluripotency of stem cell, participates in self-renew of cancer stem cell and plays important role during the initiation and development of various cancers. This study intends to investigate the expression and significance of Sox2 and Sox2 autoantibodies (Sox2-Ab) in tissue and serum of patients with non-small cell lung cancer (NSCLC).

**Methods:**

Expression of *Sox2* gene and protein was tested in 58 cases of NSCLC, 16 patients with other tumors and 20 cases of normal lung tissue specimens by quantitative PCR and immunohistochemical assay, respectively. Serum Sox2-Ab level was detected in 30 cases of NSCLC patients and 30 healthy controls by ELISA method. Clinical and pathological data from patients were collected and analyzed retrospectively.

**Results:**

Expression levels of Sox2 mRNA and protein were higher in patient with NSCLC than other groups, with statistically significant differences (*P* < 0.01), respectively. Meanwhile, Sox2 mRNA expression increased in NSCLC patients associated with histological type and tumor size. No significant differences in Sox2-Ab serum levels were found between NSCLC patients and normal subjects.

**Conclusion:**

Sox2 in NSCLC have a higher expression, which is closely related to histological type and tumor size. Sox2 might use as a potential biomarker and therapeutic target for lung cancer.

肿瘤干细胞（cancer stem cells, CSCs）在肿瘤的发生发展、侵袭衍进、术后复发、治疗疗效和肿瘤耐药等方面发挥着重要作用。干细胞核心转录因子Sox2属于*SOX*（SRY-like HMG box）基因家族成员，包含DNA结合区HMG，在维持干细胞的自我更新、多向分化及重编程等方面发挥重要作用^[1-3]^。流行病学研究^[4-7]^提示，*Sox2*基因突变、甲基化或表达异常与多种癌前病变及肿瘤的恶性生物学行为有关。同时，研究者在脑膜瘤、骨髓瘤、乳腺癌和小细胞肺癌患者的血清中检测出Sox2自身抗体（Sox2-Ab）水平增高^[8, 9]^。本研究采用实时荧光定量PCR、免疫组化及ELISA法检测非小细胞肺癌（non-small cell lung cancer, NSCLC）患者肿瘤组织Sox2表达和血清Sox2-Ab水平，旨在分析Sox2和Sox2-Ab在NSCLC的表达和意义，评价Sox2作为肺癌新的标志物及治疗靶点的价值。

## 1 材料与方法

### 1.1 标本来源与处理

收集2010年1月-2013年3月江苏省肿瘤医院和南京医科大学第一附属医院组织及血清标本，包括58例NSCLC（基于2009年国际抗癌联盟UICC肺癌TNM分期，腺癌30例和鳞癌28例，表 1）、16例其他类型肿瘤（乳腺癌4例、食管鳞癌2例、胃腺癌4例、肠腺癌3例和肝癌3例）以及20例正常肺脏的手术组织标本，术前均未经放化疗。病理（痰、胸水、肺活检及肺泡灌洗液等）确诊的肺癌患者治疗前血样30例（腺癌15例和鳞癌15例）及健康体检者血清30例。该研究得到南京医科大学第一附属医院伦理委员会同意。

**1:** 肺癌组织中Sox2 mRNA表达与临床病理因素的关系 The relation between expression of Sox2 mRNA in NSCLC

Pathologic parameter	*n*	mRNA expression	*P*
Age (year)			0.100
≤60	32	1.05±0.16	
> 60	26	1.99±0.32	
Gender			0.160
Male	36	1.63±0.23	
Female	22	1.06±0.29	
Tumor size			0.008
≤3 cm	27	0.95±0.20	
> 3 cm	31	1.90±0.25	
Histology			0.001
Adenocarcinoma	30	0.87±0.21	
Squamous carcinoma	28	2.04±0.23	
Differentiation			0.240
None or well	25	1.19±0.33	
Moderate or poor	33	1.64±0.21	
Lymph node metastasis			0.816
Negative	35	1.43±0.22	
Positive	23	1.52±0.34	
NSCLC: non-small cell lung cancer			

### 1.2 方法

#### 1.2.1 实时荧光定量PCR

按照EZNA FREE石蜡组织样本RNA提取试剂盒提取总RNA。利用逆转录试剂盒RevertAid First Strand cDNA synthesis Kit合成cDNA。基因库检索获得*Sox2*基因全长序列，利用软件Primer primier 5.0，设计上游引物：5-tggacagttacgcgcacat-3，下游引物：5-cgagtaggacatgctgtaggt-3，扩增片段长度为205 bp，引物由上海英俊公司合成。实时荧光定量PCR采用荧光染料IQ SYBR Green Supermix掺入法。

#### 1.2.2 免疫组织化学

采用SP法进行Sox2免疫组化染色。兔抗人Sox2单克隆抗体为美国CST公司产品，SP试剂盒和DAB显色剂为美国Zymed公司产品，均购自巴傲得生物技术有限公司。免疫组化染色按试剂盒操作说明进行，以微波加热法行抗原修复，用PBS代替一抗做阴性对照。光学显微镜下高倍镜观察Sox蛋白阳性部位位于肺癌细胞核，以细胞核出现广泛棕黄色和棕褐色颗粒为阳性，以未染色或淡黄色为阴性，并与荧光半定量PCR结果联合分析，400倍光镜下随机选择10个视野，以平均每个视野超过5%阳性染色细胞为阳性。

#### 1.2.3 ELISA

采早晨空腹静脉血3 mL，分离血清，置于-20 ℃冰箱待测。血清Sox2-Ab水平应用酶标仪（Bio-Rad Model680），按照ELISA检测试剂盒（上海瑶韵生物科技有限公司）说明书进行检测。

### 1.3 统计及分析

应用SPSS 17.0软件作统计学处理。阳性率的比较采用χ^2^检验或fisher确切概率法，均数的比较采用*t*检验，以*P* < 0.05为差异有统计学意义。

## 2 结果

### 2.1 Sox2 mRNA在不同组织中的表达

实时荧光定量PCR统计结果表明肺癌组织中Sox2 mRNA表达明显高于正常肺组织，前者均值是后者均值的3.93倍，二者相比差异有统计学意义（*t*=4.41, *P* < 0.001），肺癌组织最高者表达量是正常肺组织均值的24.04倍。同时，Sox2 mRNA表达高于其他肿瘤组织，其均值是后者的2.11倍，差异有统计学意义（*t*=4.38, *P* < 0.001）（图 1）。而Sox2 mRNA在其他肿瘤组织和正常肺组织中的表达水平无统计学差异（*t*=0.86, *P*=0.403）。

**1:**
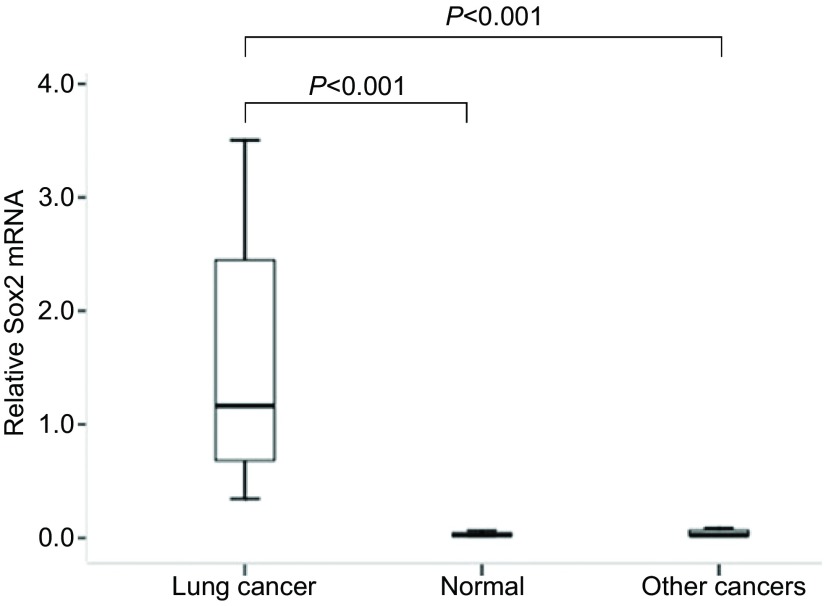
实时荧光定量PCR检测各种组织中Sox2 mRNA的表达 Expression of Sox2 mRNA in tissues by qRT-PCR

### 2.2 肺癌组织中Sox2 mRNA表达与临床病理特征之间的关系

Sox2 mRNA表达与NSCLC患者的年龄、性别、肿瘤分化程度和淋巴结转移情况无关，但与肿瘤的体积和组织学类型相关，即Sox2 mRNA在鳞癌的表达高于腺癌；Sox2 mRNA表达随肿瘤体积增高（表 1）。

### 2.3 Sox2蛋白在肺癌组织中的表达

58例患者肺癌细胞中，Sox2蛋白阳性表达39例，阴性表达19例，20例正常肺组织中Sox2蛋白均为阴性，与肺癌组织比较差异具有统计学意义（χ^2^=13.6, *P* < 0.01）（图 2）。

**2:**
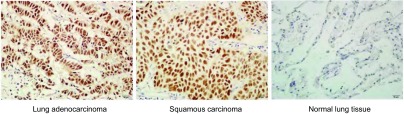
Expression of Sox2 mRNA in tissues by qRT-PCR Expression of Sox2 mRNA in tissues by qRT-PCR

### 2.4 Sox2-Ab表达水平

血清Sox2-Ab水平肺癌组（9.32±1.23）ng/mL，对照组（8.29±0.71）ng/mL，肺癌组血清Sox2-Ab均值水平高于对照组，但差异无统计学差异（*P*=0.09）。Sox2-Ab表达水平在不同年龄、性别、病理类型、分化程度和淋巴结转移的NSCLC患者间差异无统计学意义。

## 3 讨论

肺癌的发病率居高不下，死亡率居恶性肿瘤首位。其中，NSCLC约占肺癌的80%，发现时多是晚期，5年生存率不足15%^[10]^。因此，探索肺癌的病因和发病机制，寻找新的治疗靶点及标志物具有重要意义。

肿瘤干细胞系是具有干细胞性质的癌细胞，是肿瘤生长的驱动力。干细胞转录因子*Sox*基因所编码的蛋白参与了早期胚胎形成、神经发育、性别决定、细胞命运决定乃至肿瘤发生等重要的生物学过程。研究^[1]^证实，*Sox*基因家族成员之一的*Sox2*基因对维持胚胎干细胞的全能性发挥了关键作用；Sox2协同*Nanog*、*Oct3*/*4*基因维持胚胎干细胞自我更新^[2]^；同时联合*klf4*、*c-Myc*等基因能诱导成纤维细胞向多能干细胞转化^[3]^。

针对肺癌的研究提示，*Sox2*系多效性的原癌基因。Sox2诱导鳞癌标记肿瘤相关因子p63和角蛋白6表达，影响鳞癌的分化、迁移和侵袭^[4]^，肝细胞生长因子受体和Sox2扩增多见于吸烟的肺鳞癌患者^[6]^；Sox2调节A549肺腺癌侧群细胞包括c-MYC、WNT1、WNT2和NOTCH1等的246个靶癌基因的表达，维持A549等肺癌侧群细胞的肿瘤“干性”^[7, 11]^；全基因组分析研究发现，Sox2、FGFR1或MYC家族基因扩增驱动小细胞肺癌（small cell lung cancer, SCLC）的发生发展^[12, 13]^；Sox2诱导肿瘤癌信号EGFR及BCL2L1，促进肺癌细胞的增殖、存活^[14]^；Sox2还是Ⅰ期肺腺癌预后不佳的独立预测因子，且同复发风险相关^[5]^。

本研究结果显示，Sox2在NSCLC组织中有较高表达，且鳞癌高于腺癌，而在其他非肺部肿瘤及正常肺组织中表达较低，提示Sox2在NSCLC中的表达具有较高的特异性和敏感性，Sox2可作为NSCLC新的标志物。进一步分析发现，Sox2在NSCLC中的表达与肿瘤体积正相关，这可能同Sox2诱导下游EGFR、IGF-1信号，促进细胞增殖和存活有关^[14, 15]^。而Ruan等^[16]^研究也发现Sox2表达相关于T1膀胱癌肿瘤大小、肿瘤数量和级别。

鉴于Sox2的免疫源性，研究者在SCLC患者的血清中检测出Sox2-Ab，其诊断SCLC的敏感性为33%（95%CI: 27%-40%），特异性为97%（95%CI: 94%-99%），有望成为SCLC诊断的特异性生物学标志，但进一步研究提示Sox2-Ab水平对SCLC的预后无明显影响^[8]^。乳腺癌研究中，乳腺癌患者血清Sox2-Ab水平明显高于乳腺良性疾病患者和健康对照者，且Sox2-Ab水平同乳腺癌患者肿瘤分期及淋巴结转移相关，血清Sox2-Ab检测在区分良恶性乳腺肿瘤中较组织多肽抗原、癌胚抗原、糖类抗原125及糖类抗原153等肿瘤标志物更有价值^[9]^。

本研究采用常规的ELISA方法检测NSCLC患者及健康体检者血清中Sox2-Ab表达水平，并没有阳性发现，可能同样本量较小，检测方法不灵敏，以及肺腺癌患者血清样本相对较多等因素有关。另外，本研究中，其他肿瘤组织样本也以腺癌居多，如乳腺癌、胃腺癌等，可能影响了其他肿瘤的代表性，今后需扩大研究样本，进一步证实该结果。

综上，Sox2在NSCLC组织中有较高的表达，肺鳞癌高于肺腺癌，且该表达同肿瘤体积正相关。Sox2可能是参与肺癌的发生发展重要因素，有望成为肺癌新的标志物及治疗靶点。
